# Evolution of primate T-cell leukemia virus type 1 accessory genes and functional divergence of its antisense proteins

**DOI:** 10.1371/journal.ppat.1013158

**Published:** 2025-05-09

**Authors:** Osama Hussein, Mohamed Mahgoub, Takafumi Shichijo, So Nakagawa, Junko Tanabe, Hirofumi Akari, Tomoyuki Miura, Masao Matsuoka, Jun-ichirou Yasunaga

**Affiliations:** 1 Department of Hematology, Rheumatology, and Infectious Diseases, Faculty of Life Sciences, Kumamoto University, Kumamoto, Japan; 2 Laboratory of Virus Control, Institute for Frontier Life and Medical Sciences, Kyoto University, Kyoto, Japan; 3 Department of Molecular Life Science, Tokai University School of Medicine, Isehara, Kanagawa, Japan; 4 Center for the Evolutionary Origins of Human Behavior, Kyoto University, Inuyama, Aichi, Japan; 5 Laboratory of Primate Model, Institute for Frontier Life and Medical Sciences, Kyoto University, Kyoto, Japan; University of Illinois at Chicago College of Medicine, UNITED STATES OF AMERICA

## Abstract

Human T-cell leukemia virus type 1 (HTLV-1) is derived from simian T-cell leukemia virus type 1 (STLV-1), and together they form a broader category known as primate T-cell leukemia virus type 1 (PTLV-1). PTLV-1 encodes multiple proteins from overlapping open reading frames (ORFs) in the pX region. This study aims to characterize the conservation of these proteins in different PTLV-1 subtypes and their role in pathogenesis. For the first time, we report the full-length proviral sequence of an STLV-1 strain isolated from chimpanzee and African green monkey. Phylogenetic analysis reveals high conservation of the accessory proteins p12, p30, and p13 in the HTLV-1a subtype. Conversely, some African PTLV-1 subtypes exhibit loss of ORFs for p12 or p13. For Asian subtypes, simian strains often lack p12, p13, or p30 proteins, whereas human strains retain the ORFs of p30 and p13 but not p12. To assess the infectivity of a simian strain of PTLV-1 lacking ORFs for p12, p13, and p30, we constructed a molecular clone from a naturally infected Japanese macaque (Mfu: *Macaca fuscata*) and compared it with HTLV-1a. Using a reporter assay and ELISA, we found similar infectivity to Jurkat T cells; however, STLV-1 Mfu exhibited impaired infectivity in the monocytic cell line THP-1. Additionally, despite the conservation of the HTLV-1/STLV-1 bZIP factor (HBZ/SBZ) ORFs, HBZ/SBZ proteins derived from HTLV-1a and African PTLV-1 subtypes induce significantly higher activation of the TGF-β/Smad signaling pathway than those from Asian subtypes. Collectively, our findings suggest that the acquisition of the accessory proteins by PTLV-1 subtypes potentially confers an advantageous adaptation of PTLV-1 during infection in apes, including humans. Moreover, among PTLV-1 strains, HBZ/SBZ had varying degrees of activity on the TGF-β/Smad pathway; this fact underscores the complex interplay between viral proteins and host signaling pathways, possibly influencing the viral pathogenicity in different species.

## Author summary

Primate T-cell leukemia virus type 1 (PTLV-1) is a category of viruses that includes human T-cell leukemia virus type 1 (HTLV-1) and simian T-cell leukemia virus type 1 (STLV-1). Originating in monkeys, this virus has crossed species barriers and transmitted to humans on multiple occasions in Asia and Africa over thousands of years. This study characterizes the genetic diversity of different PTLV-1 subtypes and examines their association with geographical locations and host species. We found that while certain proteins (the so-called accessory proteins) are conserved in the most common human subtype (HTLV-1a subtype), some African and Asian subtypes have lost them. Interestingly, only STLV-1 strains found in chimpanzees and gorillas retain them all. Additionally, although the antisense proteins, HTLV-1 and STLV-1 bZIP factor (HBZ/SBZ) are conserved, they function differently across subtypes. Our findings suggest that the acquisition of the accessory proteins through evolution may have enhanced viral adaptability to the host, while differences in HBZ/SBZ functionality across species could influence the pathogenicity of the virus in different hosts. These findings advance our understanding of PTLV-1’s pathogenic mechanisms and underscore the need for more complete STLV-1 sequences from diverse host species and comparative infectivity studies to clarify the evolution of PTLV-1.

## Introduction

Primate T-cell leukemia virus type 1 (PTLV-1) is a delta retrovirus that exclusively infects human and simian hosts. Depending on the host, PTLV-1 is named human T-cell leukemia virus 1 (HTLV-1) or simian T-cell leukemia virus 1 (STLV-1). HTLV-1 causes adult T-cell leukemia-lymphoma (ATL) or inflammatory diseases such as HTLV-1-associated myelopathy/tropical spastic paraparesis (HAM/TSP) in approximately 5% of infected individuals [1]. PTLV-1-induced malignancies similar to ATL have also been identified in simian hosts, such as gorillas [2], baboons [3], and African green monkeys [4,5]. Previous phylogenetic and molecular clock studies have suggested that PTLV-1 originated in simian hosts in Asia, giving rise to the Asian STLV-1 subtype and its human descendant (HTLV-1c) [6–10]. Subsequently, Asian STLV-1 was introduced to Africa, where the African PTLV-1 subtypes (HTLV-1a, PTLV-1b, PTLV-1d, PTLV-1e, and PTLV-1f) emerged [11–13]. HTLV-1a (also known as cosmopolitan HTLV-1) is the most prevalent subtype and so far has only been found in humans [14].

The HTLV-1 provirus contains three structural genes (*gag*, *pol*, and *env*), two regulatory genes *(tax and rex*), and four accessory (auxiliary) genes (*p12/p8*, *p30*, *p13,* and *HTLV-1 bZIP factor* [*HBZ*]) [15–17]. The plus strand of the pX region, located downstream of the *env* gene, encodes five of these regulatory and accessory proteins from four overlapping open reading frames (ORFs): Tax (ORF-IV), Rex (ORF-III), p30 and p13 (both derived from ORF-II), and p12/p8 (ORF-I) [18,19]. Additionally, the minus strand of the provirus encodes HBZ protein [17,20,21]. HTLV-1 exemplifies the *de novo* origin of viral genes by overprinting pre-existing genes [22]. *Tax* is estimated to be the earliest gene to emerge in the pX region in the common ancestor of delta retroviruses, followed by *rex* (by overprinting the pre-existing *tax* ORF) and *HBZ* in the common ancestor of PTLV (ancestor of PTLV-1, 2, 3, and 4). Subsequently, the accessory proteins p12/p8, p30, and p13 evolved *de novo* on top of the former ORFs in the pX region in PTLV-1 [23]. The later emergence of accessory proteins (compared to Tax, Rex, and HBZ) is supported by multiple reports of some PTLV-1 strains lacking these proteins, particularly among STLV-1 strains [24–26]. Like PTLV-1, other T-cell leukemia viruses such as PTLV-2 and PTLV-3 encode putative accessory proteins in the pX region of the provirus genome, but these proteins differ from the PTLV-1 accessory proteins [27].

Earlier *in vitro* studies have shown that p12, p30, and p13 are dispensable for HTLV-1 replication and primary T-cell transformation [28–30]. However, it was shown that p12 and p30 are required for HTLV-1 infectivity *in vivo* using a rhesus macaque model, suggesting they have a crucial role in the infection of dendritic cells [31]. In addition to viral infectivity and replication, *in vitro* studies have established multiple roles of these accessory proteins in T-cell proliferation and transformation, viral latency, and immune evasion [32]. Those studies suggest that the roles of the accessory proteins may vary depending on their hosts and cell context. For example, when cross-species transmission occurs, these viral proteins may interact differently (or fail to interact) with their cellular targets in the new host species.

Most previous studies on HTLV-1 genomes have focused primarily on the HTLV-1a subtype, due to its high prevalence in humans. However, recent reports highlight the roles of accessory proteins in HTLV-1 infectivity and immune regulation [32]. Therefore, characterization of the preservation of these proteins among different PTLV-1 subtypes could provide more insight into the significance of these proteins, the virulence of different subtypes, and the evolution and interspecies transmission history of HTLV-1. Our previous investigation revealed that STLV-1 from a Japanese macaque (Mfu; *Macaca fuscata*) lacks ORFs for all three accessory proteins, consistent with a recent study reporting the absence of accessory genes in another (highly divergent) Asian STLV-1 strain [26]. We also found substantial variation among the STLV-1 and HTLV-1 subtypes regarding the presence or absence of ORFs encoding one or more of these proteins. In this study, we comprehensively characterized the potential of various strains of PTLV-1 to encode the accessory and regulatory proteins, using multiple sequence alignments and annotation of publicly available viral sequences combined with those from strains newly sequenced for this study. We analyzed the evolutionary history of these proteins, considering the PTLV-1 subtype, geographical distribution, and primate host. We also constructed a full-length STLV-1 molecular clone derived from Mfu which is deficient in the three accessory proteins, and we found that while it could replicate efficiently in a human T cell line, its replication was significantly reduced in a monocyte cell line. Finally, we tested the ability of the conserved PTLV-1 protein HBZ/SBZ from various strains to synergize with the host restriction factor APOBEC3G in activating the TGF-β/Smad signaling pathway, which promotes ATL cell proliferation.

## Results

### Genomic sequencing of STLV-1 from three different simian species

**Fig 1:**
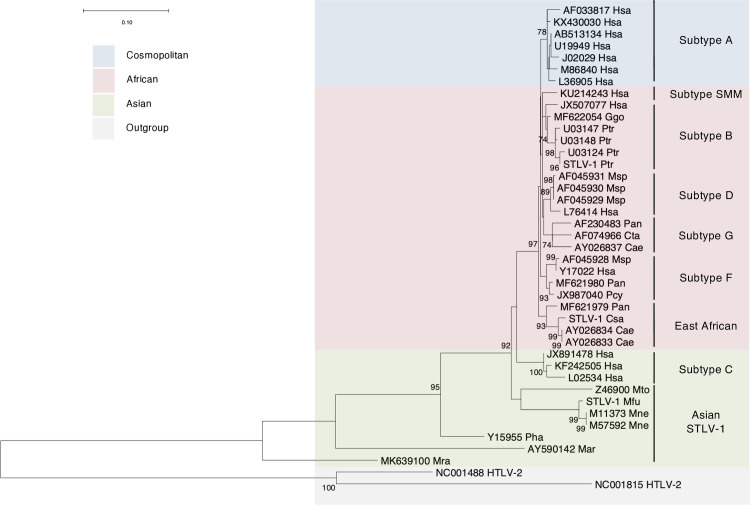
PTLV-1 phylogenetic tree and subtype assignment of STLV-1 from chimpanzee, African green monkey, and Japanese macaque. Maximum likelihood tree depicting the sequences (522 bp) corresponding to the *Env* coding region of PTLV-1. The tree comprises sequences from the publicly available database and newly sequenced STLV-1 from *Pan troglodytes* (STLV-1 Ptr), *Chlorocebus sabaeus* (STLV-1 Csa), and *Macaca fuscata* (STLV-1 Mfu). Each sequence is annotated with its NCBI accession number followed by a three-letter abbreviation denoting the genus and species (the first letter in the upper case refers to the genus, and the second and third letters in the lower case refer to the species name). The accession number for the new sequences is replaced by “STLV-1”. Fast-bootstrapping percentage values (1000 times) are indicated for each node. A scale bar for branch length is shown in the upper left of the figure. Information on the proviral sequences used for this analysis is given in S1 Table.

Initially, we sought to characterize the coding potential of the PTLV-1 accessory proteins among the various sequences available in the public database. However, only a limited number of these sequences cover the pX region downstream of the *Env* coding region (positions 6,296–8,507 in the reference HTLV-1 genome, GenBank accession number: AF033817), with no representative strain from chimpanzees, a significant source of zoonotic STLV-1 infection [33,34]. Hence, we decided to sequence STLV-1 from three different primate hosts: chimpanzees (Ptr: *Pan troglodytes*), African green monkeys (Csa: *Chlorocebus sabaeus*), and Japanese macaques (Mfu: *M. fuscata*). To this end, we extracted genomic DNA from infected cells: the ChM1141 cell line from a chimpanzee [35], the GM0650 cell line from an African green monkey [35], and primary PBMCs from a naturally infected Japanese macaque [36], and determined the full-length proviral sequences. Next, we determined the subtypes of the three newly sequenced proviruses (in addition to retrieved sequences of the proviral *Env* region) by performing multiple sequence alignment of the *Env* region sequence from these proviruses alongside other strains with a known subtype from previous studies (strains summarized in S1 Table). Our analysis revealed that STLV-1 Ptr clusters with PTLV-1 subtype B, whereas STLV-1 Csa clusters with East African PTLV-1. As expected, STLV-1 Mfu clusters with the Asian STLV-1 strains (Fig 1).

### Characterization of coding potential for the accessory proteins (p12, p30, and p13) in PTLV-1

**Fig 2:**
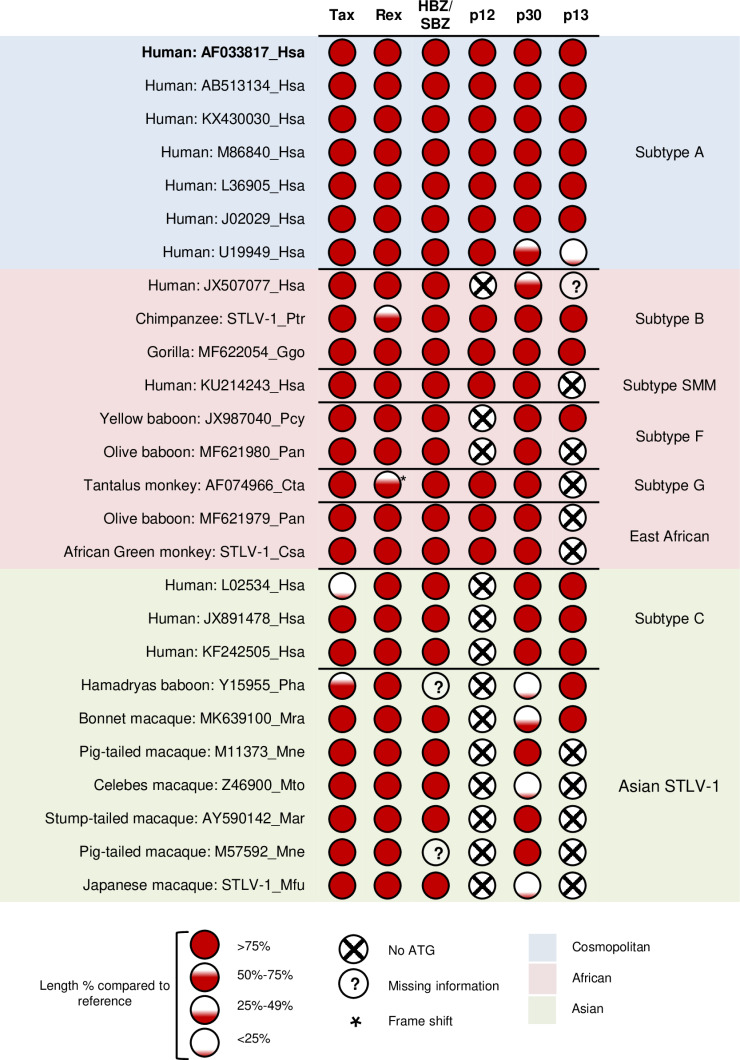
Coding potential of accessory and regulatory proteins in PTLV-1. Analysis of the coding potential of pX region ORFs for the accessory and regulatory proteins in various HTLV-1/STLV-1 strains for which a full/nearly full pX region sequence is available. HTLV-1 subtype A (AF033817 Hsa) was used as the reference. The analysis accounted for the presence/absence of the initiation codon and the predicted length of the protein. Generally, the amino acid identity was considered relatively high (>75%) in comparison to reference proteins in HTLV-1a whenever an ORF was predicted to be conserved (S1, S2, and S3 Figs and S3 Table). Exons were defined by known splicing sites in HTLV-1 subtype A**.** The conservation of splicing acceptor/donor sites in a particular strain was not considered when assembling spliced mRNA and predicting ORFs. However, the nucleotides aligned to the reference’s splicing sites for each strain are shown in S3 Table. Information on the proviral sequences used for this analysis is given in S2 Table.

Next, we analyzed the potential of the publicly available PTLV-1 strains and our newly sequenced strains to encode the regulatory and accessory proteins (S2 Table). First, we performed multiple sequence alignment using the sequence from an HTLV-1 subtype A strain that contains ORFs for all proteins as the reference genome (GenBank accession number: AF033817). We then predicted a protein to be encoded if it has an ATG triplet corresponding to the initiation codon (ATG) in the reference genome, and we noted the location of nonsense mutations that resulted in predicted truncation of the protein (Fig 2 and S3 Table). We then examined the degree to which the accessory and regulatory proteins are preserved in the various strains (Fig 2 and S1–S3 Figs).

We found that the cosmopolitan (HTLV-1a) strains exhibited a very high level of preservation of accessory protein ORFs with very few exceptions reported previously. For instance, the HTLV-1 ATL-YS strain featured a premature stop codon due to a TGG to TAG mutation (tryptophan to stop codon), resulting in prematurely truncated mutants of p30 (165 aa) and p13 (10 aa), compared to 241 aa and 87 aa, respectively, in the reference sequence [37]. Also, Furukawa and others identified a premature termination codon in p12 at aa position 87 in seven HAM/TSP patients (compared to 99 aa in the reference sequence) [25]. However, it remains unclear whether these truncated mutants arise as a result of disease-associated progression in ATL or HAM/TSP, or represent evolutionary losses of these ORFs.

In contrast to the cosmopolitan HTLV-1a subtype, African and Asian PTLV-1 subtypes exhibited a broad range of variation (Fig 2 and S1–S3 Figs, S3 Table). In African PTLV-1 subtype B, the two simian strains, from chimpanzee (Ptr) and gorilla (Ggo), encoded all three accessory proteins (p12, p30, and p13), while the human strain (Hsa: *Homo sapiens*) had p30 and p13 (25% and 97% identity to the reference, respectively) but lacked ATG for the p12 ORF. In African PTLV-1 subtype F from yellow baboon (Pcy: *Papio cynocephalus*) and olive baboon (Pan: *Papio anubis*), the p12 ORF was also lost, while the p30 ORF was preserved as it was in all other African strains of PTLV-1. The yellow baboon (Pcy) also had the p13 ORF, but the olive baboon (Pan) lacked it. In African PTLV-1 subtype G and East African PTLV-1 from tantalus monkeys (Cta: *Chlorocebus tantalus*), olive baboons (Pan), and African green monkeys (Csa), p12 and p30 were encoded in all three strains, while p13 was not encoded in any strain due to a lack of ATG. In our analysis, we also included one strain isolated from a human patient with HAM/TSP who was born in Liberia [38]; this strain was closely related to an African STLV-1 strain from sooty mangabey (smm). Interestingly, this strain maintained conserved ORFs for p12 and p30, but not for p13, mirroring the pattern observed in African STLV-1 subtypes G and East African PTLV-1. Finally, in our alignment, all known Asian PTLV-1 subtypes, both human and simian, were found to lack p12 expression owing to the absence of ATG in the p12 ORF. While the Asian human subtype (HTLV-1c) encodes p30 and p13, its simian counterparts from Asia exhibited variable coding potential for these two accessory proteins, either due to the absence of an initiation codon or the presence of a premature stop codon resulting in very short predicted proteins (Fig 2). Overall, compared to cosmopolitan HTLV-1a, African PTLV-1 strains showed higher conservation rate, sequence identity, and length coverage of the accessory proteins than Asian PTLV-1 strains (S4 Fig and S3 Table).

The above findings are in agreement with a previous report [26], which also examined the conservation of accessory proteins in PTLV-1. While the previous study focused solely on p12, p13, and p30, here we expand the analysis to include HBZ/SBZ, Tax, and Rex as well. Unlike p12, p30, and p13, the other three proteins encoded in the pX region (Tax, Rex, and HBZ) demonstrated high overall conservation, in agreement with their proposed important roles in viral replication and pathogenesis. Furthermore, we examined the conservation of the splice donor (GT) and acceptor (AG) sites for alternative splicing products of the plus and minus strands in the pX region (S3 Table). Overall, splice donor sites were highly conserved, while splice acceptor sites were also conserved, though to a lesser degree. Our analysis focused solely on sites corresponding to those in the reference HTLV-1a genome, and we could not rule out the possibility of alternate site usage for splicing. For instance, although in HTLV-1c, the translation start codon for p12 is absent, it has been proposed that HTLV-1c compensates for this by utilizing alternative splice sites in the *p12/p8* pre-mRNA. This mechanism produces a doubly spliced transcript with an intact translation start codon in exon 1 of *rex* mRNA, resulting in a novel putative 16 kDa protein (p16) [39]. By analogy, it is plausible that alternative splicing or other mechanisms could give rise to functional accessory proteins, such as for p30 and p13, in the absence of canonical ORF structures. Finally, using the RDP software, no recombination signatures were detected in the pX regions across all subtypes (summarized in S4 Table).

### Truncated p12 encoded by STLV-1 Ptr with a premature stop codon can still downregulate MHC-I

Our analysis revealed that among the non-cosmopolitan PTLV-1 strains (African and Asian subtypes, including human and simian strains), the subtype B STLV-1 strains from chimpanzees (STLV-1 Ptr) and gorillas (STLV-1 Ggo) were the only strains that appeared to encode all three accessory proteins (p12, p30, and p13). It is noteworthy, however, that in these strains, p12 had a premature stop codon after 87 amino acid residues (87% of full-length; S3 Table and S1 Fig) – a finding that was consistent with a previous study [24]. A similar truncating mutation was found in one HTLV-1a strain from an ATL patient [40]. Additionally, a premature termination codon at the same position has been reported in patients with HAM/TSP, and the virus with this mutation was transmissible to family members [25]. Two alternative possibilities could explain these observations: p12 provides selective advantages for infectivity and remains functional in the truncated form, or p12 does not provide any selective advantages for infectivity at all, regardless of its length. We argue in favor of the former possibility because of the high conservation of the p12 ORF observed in HTLV-1a. However, it remains unknown whether truncating p12 at this position impairs the pathogenicity of HTLV-1/STLV-1 *in vivo*. Therefore, we explored the functionality of this truncated p12 *in vitro*.

**Fig 3:**
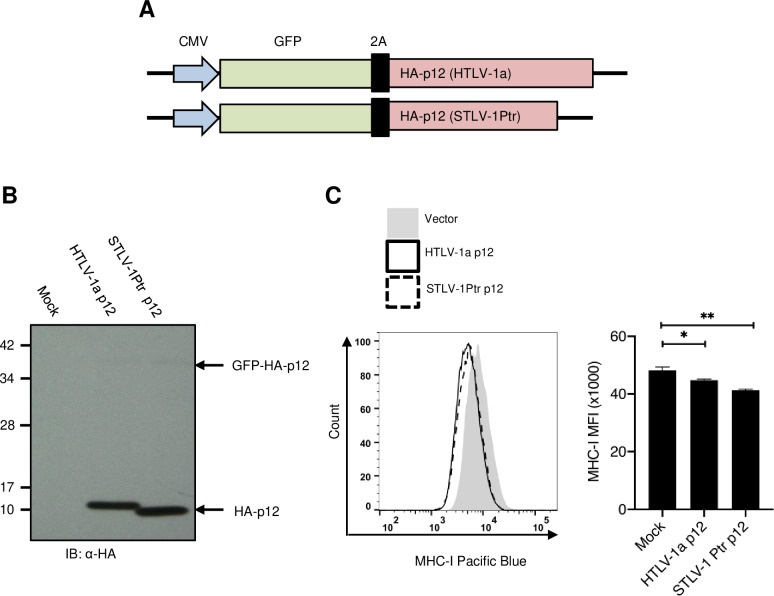
Effect of p12 on MHC-I surface expression in Jurkat Cells. **(A)** Schematic illustration of the p12 expression constructs. **(B)** Western blot analysis of HA-tagged p12 (from HTLV-1a or STLV-1 Ptr) showing expression and successful cleavage of the 2A peptide. **(C)** Flow cytometry analysis of MHC-I surface expression in GFP-positive viable Jurkat cells 5 days post-electroporation with p12-expressing plasmids or an empty vector. *Left*, representative histogram showing MHC-I surface expression level. *Right*, median fluorescence intensity (MFI) of MHC-I. Error bars represent SD for three independent triplicates. P-values were determined using unpaired t-test, * P ≤ 0.05; ** P ≤ 0.01.

In a previous study, p12 was identified as a binding partner of the major histocompatibility complex class I heavy chain (MHC-I-Hc). This binding leads to the disruption of MHC-I trafficking and downregulation of its surface expression [41]. We questioned whether the truncated version of p12 from ape STLV-1 retained this MHC-I downregulating function. To address this question, we constructed a 2A peptide-based bicistronic vector expressing GFP fluorescent marker and HA-tagged p12 cDNA cloned from HTLV-1a or STLV-1 Ptr (Fig 3A). As the HA-tag was fused to the N-terminus of p12, this approach allows the detection of p12 but not p8, the cleavage product of p12. These plasmids were transfected into HEK293T cells, and the expression of cleaved p12 protein was confirmed by immunoblotting (Fig 3B). Truncated p12 encoded by STLV-1 Ptr exhibited faster migration owing to its premature stop codon. To assess the effect of p12 on MHC-I surface expression, we electroporated these plasmids into Jurkat cells and measured MHC-I surface expression in the GFP-positive cells using flow cytometry. Both p12 proteins from HTLV-1a and STLV-1 Ptr were similarly effective in downregulating MHC-I expression (Fig 3C). This result indicated that the truncated p12 encoded by STLV-1 Ptr is functionally similar to its human counterpart, at least in terms of MHC-I downregulation.

### An STLV-1 infectious molecular clone from a Japanese macaque

**Fig 4:**
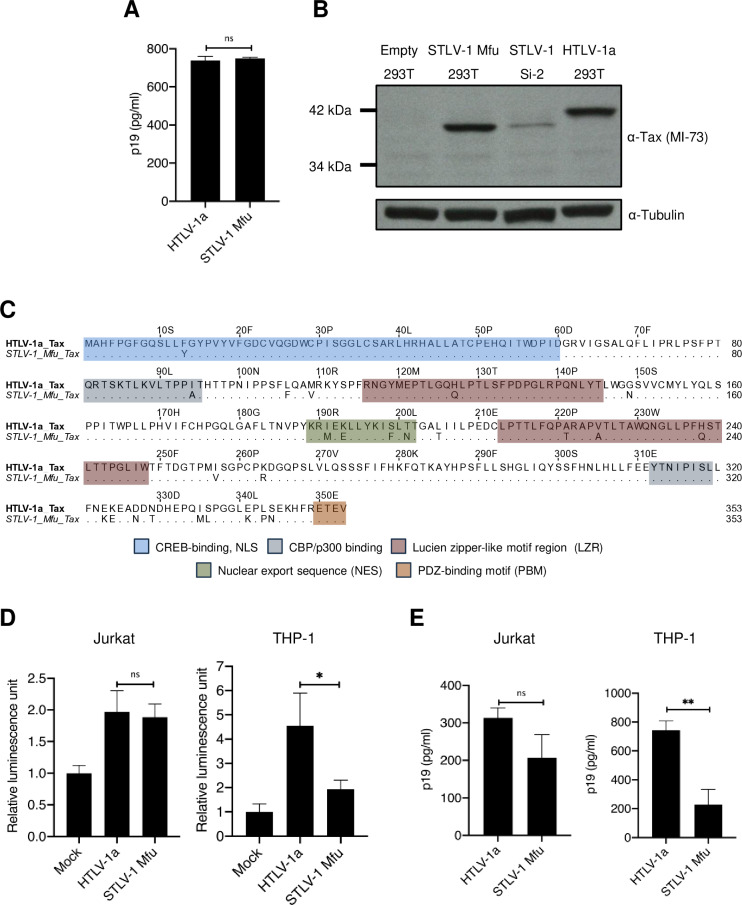
STLV-1 Mfu infectivity compares to that of HTLV-1a in T cells but not in monocytes. **(A)** Titration of virus production from an HTLV-1a or STLV-1 Mfu full-length molecular clone transfected into HEK293T cells. Cell supernatant was collected and p19 levels were measured using ELISA. **(B)** Western blot analysis depicting Tax expression in HEK293T cells transfected with HTLV-1a or STLV-1 Mfu full-length molecular clone along with the naturally STLV-1 Mfu-infected cell line (Si-2). **(C)** Multiple sequence alignment of the Tax coding sequence from HTLV-1a and STLV-1 Mfu. Key functional domains are highlighted. **(D)** Jurkat or THP-1 cells were co-cultured with virus-producing HEK293T cells for 48 hours, and the virus infectivity was measured by LTR reporter luciferase assay. **(E)** HTLV p19 antigen ELISA measuring viral production in Jurkat and THP-1 cells following co-culture with HEK293T cells transfected with HTLV-1a or STLV-1 Mfu full-length molecular clones. Three independent experiments were performed. Error bars represent SD for three replicas. P-values were calculated using unpaired t-test. * P ≤ 0.05; ** P ≤ 0.01; ns: non-significant.

We were still intrigued by the fact that the only simian strains that preserved all three accessory gene ORFs found in the human cosmopolitan strain HTLV-1a were the strains found in our closest relatives, chimpanzees and gorillas. We next asked whether naturally occurring STLV-1 lacking all three accessory proteins (p12, p13, and p30) could replicate efficiently in human cells. To this end, we constructed a full-length molecular clone of STLV-1 by amplifying a provirus from PBMCs collected from an infected Japanese macaque (Mfu) [36]. Multiple overlapping fragments were amplified from the proviral DNA and inserted into the pCR-Blunt II backbone vector through sequential cloning steps, utilizing unique restriction sites inside the provirus sequences. Subsequently, we verified the full-length clone by sequencing. To validate the infectivity of this molecular clone, we transfected it into HEK293T cells and measured virus production in the supernatant using a p19 ELISA. The p19 titer from our new STLV-1 Mfu full-length molecular clone was almost equal to that produced by the HTLV-1a full-length molecular clone pX1MT-M (plasmid vector containing a full-length HTLV-1a provirus and producing replication-competent viral particles) [42] (Fig 4A). Additionally, we verified Tax expression by the immunoblotting of HEK293T cell lysates, and again, equal levels of Tax were produced by the STLV-1 and HTLV-1 molecular clones (Fig 4B, lanes 2 and 4). Unexpectedly, Tax from STLV-1 migrated faster than Tax from HTLV-1, even though the protein alignment showed an equal number of amino acid residues for Tax from both viruses, with 92% identity (Fig 4C). Moreover, we previously reported that the splicing junctions for Tax cDNA in STLV-1 Mfu and HTLV-1a are similar, excluding the possibility of alternative splicing junctions for STLV-1 Mfu Tax cDNA [36]. To further validate this observation, we checked STLV-1 Tax expression from a naturally infected cell line (Si-2) [43] and observed the same faster migration of STLV-1 Tax compared to HTLV-1 Tax (Fig 4B, lanes 3 and 4). This difference in migration may stem from differing posttranslational modifications of Tax.

### Compared to HTLV-1a, STLV-1 Mfu infectivity is reduced in monocytes but not in T cells

Early investigations into the functions of the p12 and p30 accessory proteins showed that they were dispensable for HTLV-1 replication in T cells *in vitro* [28]. However, subsequent studies using HTLV-1 knockout mutants revealed the essential roles of these accessory proteins in virus replication within dendritic cells and some myeloid cell lines, such as THP-1 monocytes [31,44]. Thus, we questioned whether naturally occurring STLV-1 Mfu that lacks all three accessory proteins (p12, p13, and p30) would replicate differently in monocytes and T cells *in vitro* than HTLV-1a, which expresses all accessory proteins. To address this question, we compared the infectivity of HTLV-1a and STLV-1 Mfu in Jurkat T cells and THP-1 monocytic cell lines using a luciferase assay after cell-to-cell infection. Initially, we transfected donor HEK293T cells with either the HTLV-1a or the STLV-1 Mfu full-length molecular clone plasmid. Next, the recipient cells (Jurkat or THP-1 cells) were transfected with reporter plasmids expressing firefly luciferase under the control of the LTR promoter. Finally, the donor and recipient cells were co-cultured for 48 hours. The results showed comparable luciferase activity in Jurkat cells after infection with STLV-1 Mfu or HTLV-1a (p = 0.7) (Fig 4D, left panel). However, in THP-1 cells, the luciferase activity was significantly lower after the infection with STLV-1 Mfu than with HTLV-1a (p = 0.03) (Fig 4D, right panel). Consistent with these findings, HTLV p19 ELISA measurements of viral production in Jurkat and THP-1 cells revealed a similar trend (Fig 4E). These results suggest that the lack of accessory proteins in STLV-1 Mfu selectively impairs viral replication in THP-1 cells, but not in Jurkat cells.

### Functional divergence of HBZ/SBZ

**Fig 5:**
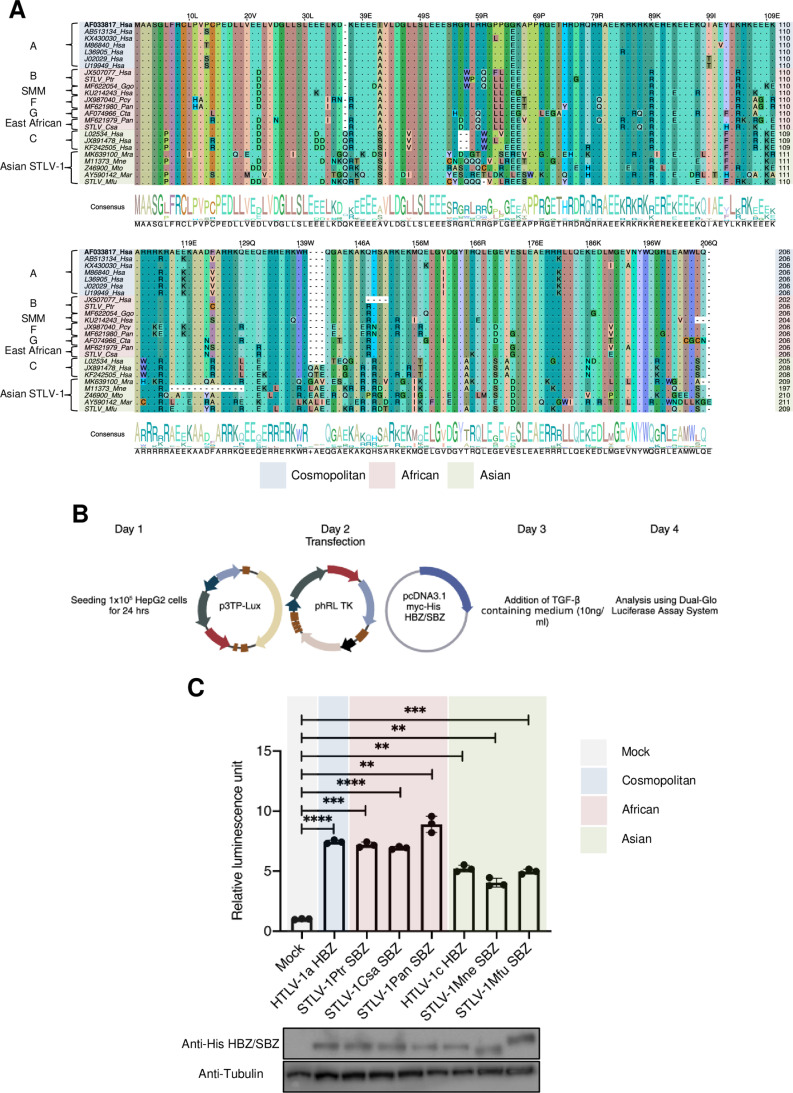
Sequence conservation but functional divergence of HBZ/SBZ. **(A)** Multiple sequence alignment of the translated nucleotide sequences of HBZ/SBZ from different primate species. Sequences were aligned using MUSCLE, and visualized using Jalview software. **(B)** Schematic illustration of the TGF-β luciferase activity assay (Created in https://BioRender.com). **(C)** Luciferase activity of 3TP-Lux under the control of a TGF-β responsive element in cells expressing HBZ/SBZ proteins derived from different PTLV-1 strains. Hsa HTLV-1a: human (subtype A), Ptr: chimpanzee, Pan: olive baboon, Csa: African green monkey, Hsa HTLV-1c: human (subtype C), Mne: pig-tailed macaque, and Mfu: Japanese macaque. The experiment was performed in triplicate; representative data is shown. Error bars represent SD for three replicas. P-values were determined using unpaired t-test. ** P ≤ 0.01; *** P ≤ 0.001; **** P ≤ 0.0001.

Our analysis demonstrated that although the conservation of the p12/p8, p30, and p13 ORFs is variable across PTLV-1 subtypes, Tax, Rex, and HBZ/SBZ ORFs are highly conserved. One would therefore expect that their functions are critical and would be preserved across all subtypes. To test this hypothesis, we examined the spliced isoform of HBZ/SBZ (Fig 5A). Multiple sequence alignment of HBZ/SBZ sequences revealed that the Asian STLV-1 subtype generally has a longer SBZ sequence (209–211 amino acids) compared to cosmopolitan HTLV-1a (206 amino acids) and the African subtypes (202–206 amino acids), whereas HTLV-1c falls between the two groups (205–208 amino acids). An exception was noted in the pig-tailed monkey (Mne: *Macaca nemestrina*), which exhibited a shorter SBZ sequence of 197 amino acids owing to an internal deletion of 13 amino acids (Fig 5A).

Previously, we reported that HBZ protein from cosmopolitan HTLV-1a activates the TGF-β/Smad signaling pathway, a crucial pathway for the proliferation of ATL cells, more robustly than does SBZ protein from an Asian STLV-1 strain found in Japanese macaques [45]. To extend these findings to additional strains of PTLV-1, we assessed the activation of the TGF-β/Smad signaling pathway by HBZ/SBZ proteins derived from PTLV-1 strains from chimpanzee (Ptr; STLV-1b), African green monkey (Csa; East African STLV-1), olive baboon (Pan; STLV-1f), human (Hsa; HTLV-1c), and pig-tailed macaque (Mne; Asian STLV-1) alongside HBZ from HTLV-1a (Hsa; cosmopolitan) and SBZ from Japanese macaque (Mfu; Asian STLV-1). To this end, we used a luciferase activity assay with 3TP-Lux, controlled by a TGF-β-responsive element, in HepG2 cell lines expressing HBZ/SBZ (Fig 5B). Our results revealed that the TGF-β activation activity of HBZ/SBZ varied across subtypes, with the cosmopolitan and African versions tending to have somewhat more of this activity than the Asian ones (Fig 5C).

These findings suggest that, despite the conservation of the HBZ/SBZ ORFs across different PTLV-1 subtypes, the HBZ/SBZ protein exhibits subtype-specific differences in its ability to activate the TGF-β/Smad signaling pathway, at least in human cells. Whether this pattern extends to HBZ/SBZ in cells from its natural host species is not yet known. Nevertheless, these observations remain very relevant to understanding the pathogenesis of these strains in humans.

### APOBEC3G isoforms manifest variable lengths in different primate species

**Fig 6:**
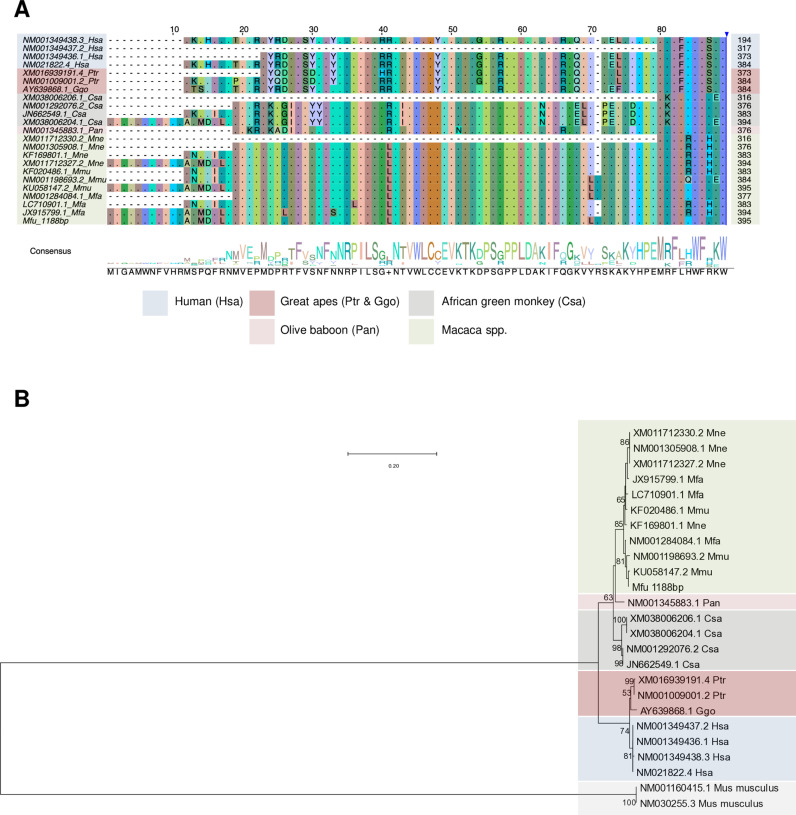
Multiple sequence alignment and phylogenetic analysis of APOBEC3G. **(A)** Multiple sequence alignment of the translated nucleotide sequences for APOBEC3G from various primate species. The first 90aa are shown here; the full-length sequences are shown in S5 Fig. Sequences from the publicly available database and an in-house sequenced Japanese macaque APOBEC3G (Mfu_395aa) were aligned using MUSCLE and visualized using Jalview software. **(B)** The maximum likelihood tree of the APOBEC3G gene comprising the full-length sequences shown in S5 Fig along with *Mus musculus* APOBEC3 protein as an outgroup. Each sequence is annotated with its NCBI accession number followed by a three-letter abbreviation denoting the genus and species. For the new sequences, the accession number is replaced by the genus and species abbreviation. Fast-bootstrapping percentage values (1000 times) are indicated for each node. A scale bar for the branch length is shown in the upper left of the figure.

Because HBZ is known to interact with APOBEC3G in important ways, we were interested in learning the degree to which this function of HBZ is preserved across subtypes. APOBEC3G, a cytidine deaminase from the APOBEC family, plays a crucial role in antiviral defense by introducing mutations in the proviral genome. This occurs through the deamination of cytosine residues to uracil during reverse transcription. In HIV-1, this defense mechanism is countered by the viral protein Vif, which targets APOBEC3G for degradation [46–49]. Similarly, SBZ from STLV-1 found in Japanese macaques (STLV-1 Mfu SBZ) inhibits the deaminase activity of simian APOBEC3G, whereas HTLV-1a HBZ does not inhibit that of human APOBEC3G. However, HTLV-1a HBZ protein still interacts with APOBEC3G – and synergistically enhances APOBEC3G-induced activation of the TGF-β/Smad pathway, promoting the proliferation of T cells. This cooperative interaction is notably absent in STLV-1 Mfu SBZ, which does not synergize with simian APOBEC3G to enhance TGF-β signaling [45,50]. Intrigued by these species-specific differences, we analyze the APOBEC3G sequences in various primates. We retrieved the sequences of APOBEC3G for humans (Has), gorillas (Ggo), chimpanzees (Ptr), African green monkeys (Csa), olive baboons (Pan), and three macaque species: pig-tailed macaque (Mne), rhesus macaque (Mmu: *Macaca mulatta*), and cynomolgus macaque (Mfa: *Macaca fascicularis*) from the public databases and compared them alongside our in-house sequence of APOBEC3G from Japanese macaque (Mfu) (summarized in S5 Table). Multiple sequence alignment of APOBEC3G proteins (Fig 6A and S5 Fig) revealed a noticeable pattern: humans, gorillas, chimpanzees, and baboons possess APOBEC3G protein isoforms with a maximum length of 384aa, whereas macaque species and African green monkeys can exhibit either the 395/394 aa isoform or a shorter one (S5 Fig). To better understand the evolutionary relationships between these isoforms of APOBEC3G, we conducted multiple sequence alignment and phylogenetic analysis. As anticipated, the results showed that human, gorilla, and chimpanzee APOBEC3G sequences are closely related. On the other hand, African green monkeys, baboons, and the macaque species each fall into a separate clade (Fig 6B). These findings suggest that the differences in the APOBEC3G among these primates may influence its role in TGF-β/Smad pathway activation and, consequently, the pathogenicity of PTLV-1.

### APOBEC3G isoforms showed variable capacity to activate the TGF-β/Smad signaling pathway

**Fig 7:**
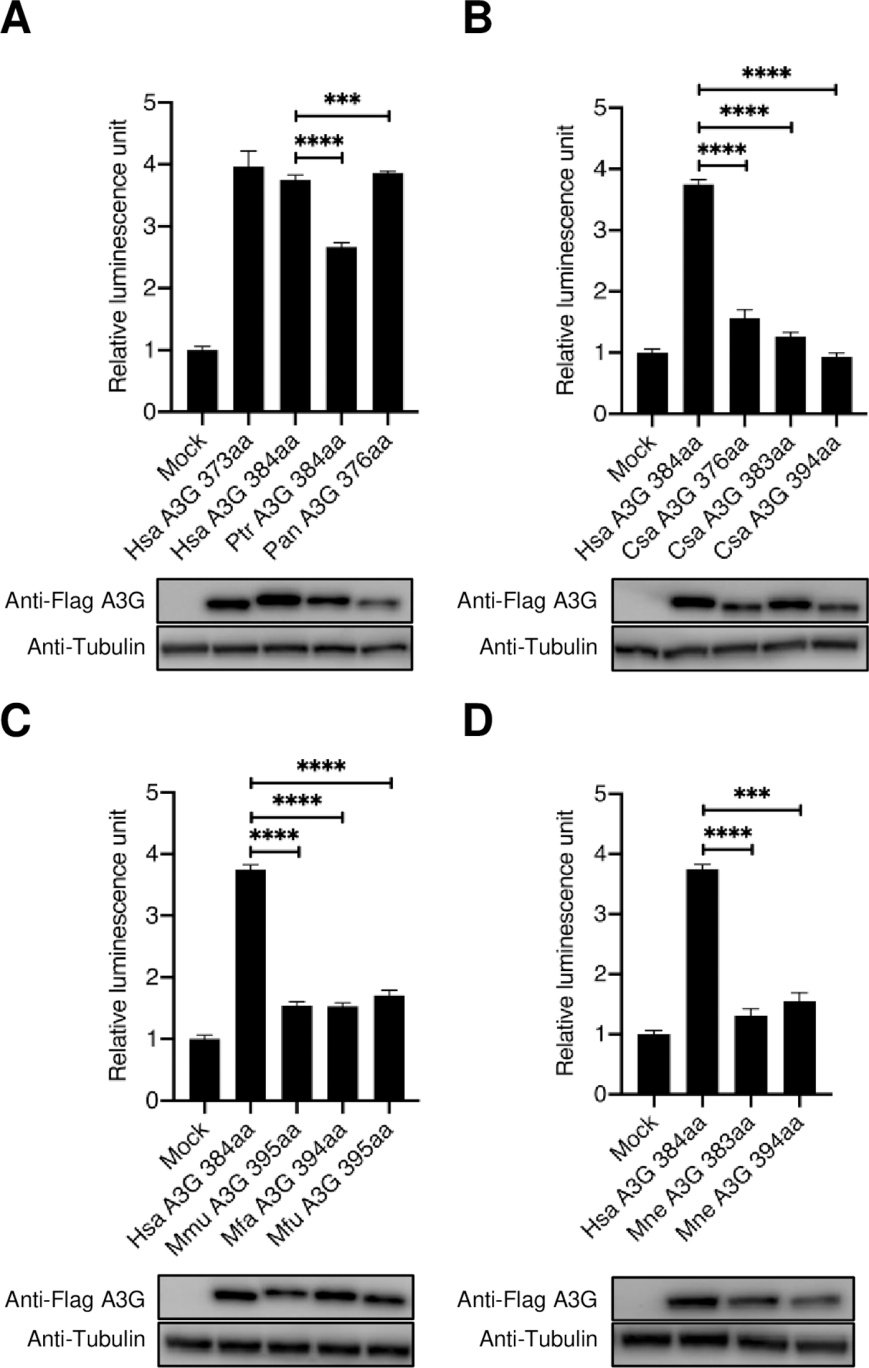
TGF-β **/Smad signaling pathway activation by primate APOBEC3G.** Luciferase activity of 3TP-Lux under the control of a TGF-β responsive element in cells expressing different APOBEC3G isoforms derived from different primate species. Human APOBEC3G (Hsa A3G 384aa) was used as a control. **(A)** Luciferase activity in cells expressing human APOBEC3G (Hsa A3G 373aa and Hsa A3G 384aa), chimpanzee APOBEC3G (Ptr A3G 384aa), and olive baboon APOBEC3G (Pan A3G 376aa). **(B)** Luciferase activity in cells expressing Hsa A3G 384aa and African green monkey APOBEC3G (Csa A3G 376aa, 383aa, and 394aa). **(C)** Luciferase activity in cells expressing Hsa A3G 384aa, rhesus macaque APOBEC3G (Mmu A3G 395aa), crab-eating macaque APOBEC3G (Mfa A3G 394aa), and Japanese macaque APOBEC3G (Mfu A3G 395aa). **(D)** Luciferase activity in cells expressing Hsa A3G 384aa and pig-tailed macaque APOBEC3G (Mne A3G 383aa and 394aa). Each experiment was performed in triplicate; representative data are shown. Error bars indicate SD for three replicas. P-values were determined using unpaired t-test. *** P ≤ 0.001; **** P ≤ 0.0001.

We next examined the functional capacity of each of these APOBEC3G isoforms to activate the TGF-β/Smad pathway. To this end, we performed a luciferase reporter activity assay using 3TP-Lux in HepG2 cells expressing APOBEC3G from various species. The results revealed that human APOBEC3G isoforms 2 and 1 (Hsa A3G 373 aa and 384 aa, respectively) exhibited high TGF-β/Smad activation activity, as did the APOBEC3G proteins from olive baboon (Pan A3G 376 aa) and chimpanzee (Ptr A3G 384 aa) (Fig 7A). For African green monkeys, the shortest isoform (Csa A3G 376 aa) demonstrated higher activity compared to the longer isoforms (Csa A3G 383 aa and 394 aa) (Fig 7B). The APOBEC3G isoforms from macaque species demonstrated low or no TGF-β/Smad activation capacity at all (Fig 7C and 7D).

**Fig 8:**
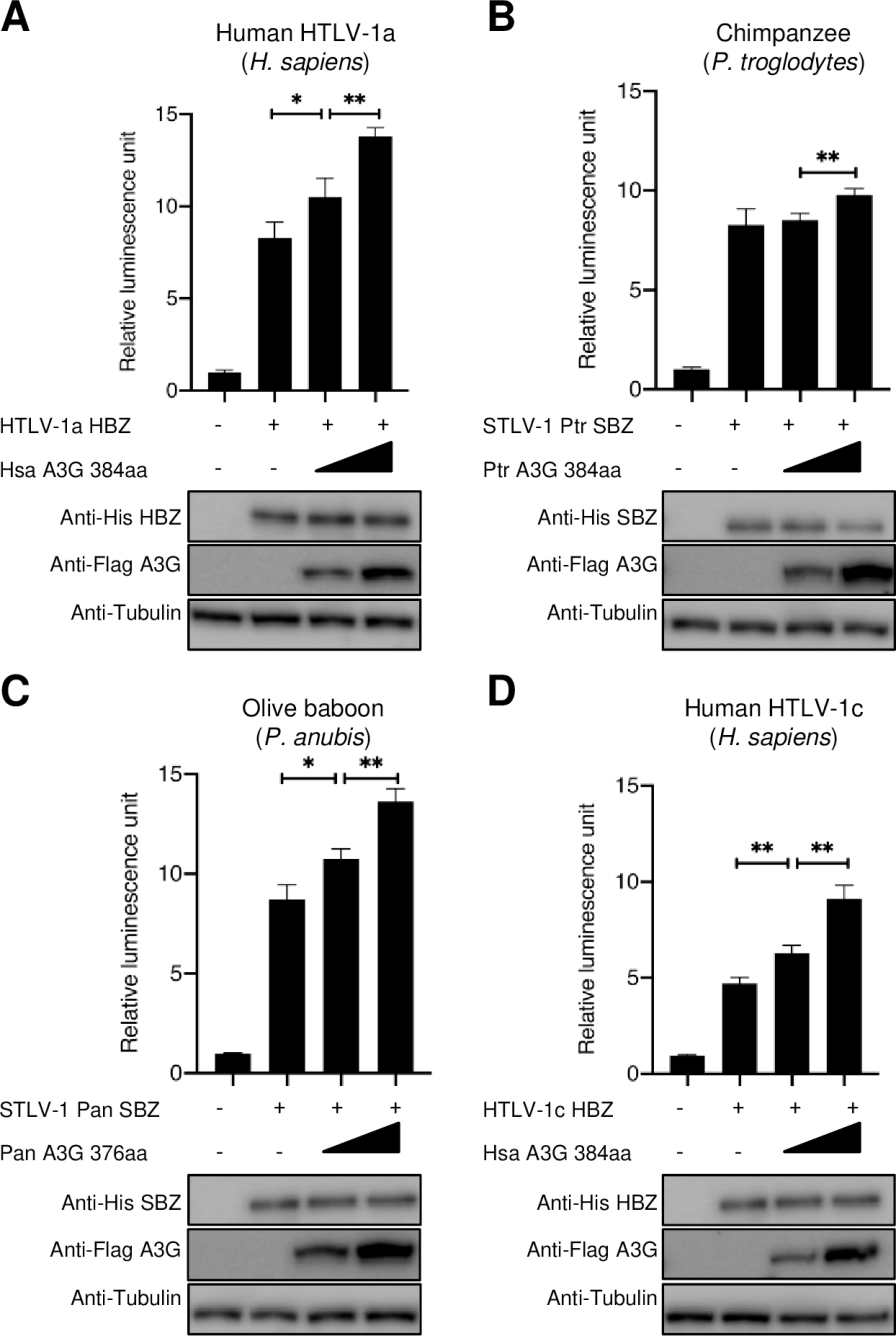
Synergistic activation of the TGF-β **/Smad signaling pathway by SBZ/HBZ and primate APOBEC3G.** Luciferase activity of 3TP-Lux under the control of a TGF-β responsive element in cells expressing HBZ/SBZ and increasing concentrations of APOBEC3G derived from four different primate species. In each case, HBZ/SBZ from PTLV-1 isolated from a particular primate species is paired with APOBEC3G from that same species. **(A)** Luciferase activity in cells expressing HTLV-1a HBZ and human APOBEC3G 384aa **(B)** Luciferase activity in cells expressing STLV-1 Ptr SBZ and chimpanzee APOBEC3G 384aa. **(C)** Luciferase activity in cells expressing STLV-1 Pan SBZ and olive baboon APOBEC3G 131aa. **(D)** Luciferase activity in cells expressing HTLV-1c HBZ and human APOBEC3G 384aa. Each experiment was performed in triplicate; representative data are shown. Error bars indicate the SD for three replicas. P-values were determined using unpaired t-test. *P ≤ 0.05; **P ≤ 0.01.

Finally, we tested whether HBZ/SBZ together with APOBEC3G from the relevant primate host species could synergistically activate the TGF-β/Smad signaling pathway. We focused on chimpanzee and olive baboon, because their APOBEC3G proteins demonstrated a strong ability to activate the TGF-β/Smad pathway in the above experiments. Like HBZ from human strains of HTLV-1a/c, SBZ from olive baboon strain could significantly enhance TGF-β/Smad signaling when co-expressed with the species-appropriate APOBEC3G (Fig 8A–D). Taken together, these findings highlight the significant role of APOBEC3G in modulating TGF-β/Smad signaling pathway activation, and the preservation of this function across different primate hosts. The varying degrees of APOBEC3G proteins to activate the TGF-β/Smad pathway synergistically with HBZ/SBZ may ultimately influence the PTLV-1 pathogenicity in different species.

## Discussion

In this study, we report for the first time the full genomic sequence of an STLV-1 strain isolated from chimpanzees and African green monkey, and we calculate its position on the phylogenetic tree of PTLV-1, showing that it belongs to subtype B and East African PTLV-1, respectively. Our analysis revealed no evidence suggesting that STLV-1 from chimpanzees or gorillas is a direct ancestor of HTLV-1a. Hence, the simian ancestor of cosmopolitan HTLV-1 remains to be identified.

Phylogenetic analysis revealed a high degree of preservation of the ORFs encoding the accessory proteins among PTLV-1 strains isolated from HTLV-1a, STLV-1Ptr, and STLV-1Ggo, but not those isolated from monkeys. For Asian PTLV-1, it seems likely that p12 failed to be preserved and hence is lacking in both human and simian strains. However, since p12 ORF is highly preserved in the HTLV-1a subtype and provides a selective advantage for the HTLV-1 strains, its absence in HTLV-1c is likely due to the loss of the ORF in the simian ancestor of HTLV-1c, rather than occurring after cross-species transmission. This loss is proposed to be compensated by the production of a novel p16 protein through alternative splicing [39]. If this is the case, similar mechanisms could exist for other accessory proteins, p30 and p13 highlighting the potential for functional adaptability in the absence of canonical ORF structures. However, the point remains that the other accessory proteins, p30 and p13, are best preserved among human strains, and not well preserved among strains from monkeys. Overall, African PTLV-1 featured a higher preservation rate for the accessory proteins’ ORFs compared with Asian PTLV-1. Moreover, since in Africa, the simian-human interspecies barrier has been crossed multiple times [12], this fact may contribute to the overlapping preservation pattern of accessory proteins between African subtypes of HTLV-1 and STLV-1. In contrast, previous analyses have suggested that a single cross-species transmission event took place from Asian STLV-1 to give rise to HTLV-1c [51]. Thus, the observed differences in the presence of accessory protein ORFs between Asian STLV-1 and HTLV-1c likely reflect evolutionary divergence, potentially due to the loss of p13 and p30 ORFs in the macaque lineage following the single cross-species transmission event that gave rise to HTLV-1c. On the other hand, it is possible to hypothesize that African PTLV-1 shares a common ancestor in which all three accessory proteins were preserved. That ancestor was likely a strain that infected apes, including humans but not monkeys since preserving these proteins was more advantageous to human/ape progeny strains than to monkey strains. Later on, one or more of these accessory proteins were lost due to nonsense mutations in some species. This is particularly likely if they did not provide a selective advantage in the new species after crossing a species barrier, as they were no longer useful, and there was no selection pressure to maintain them. However, this study was limited by the scarcity of available STLV-1 sequences encompassing the pX region that encodes the accessory proteins. Therefore, to gain a better insight into the evolution of cosmopolitan HTLV-1, sequencing of more STLV-1 strains from diverse natural simian hosts is imperative. Moreover, obtaining additional sequences from chimpanzee and gorilla hosts in particular will strengthen our current findings on the preservation of the p12, p30, and p13 ORFs in these two primate hosts.

Viruses can cross interspecies barriers by evolving adaptive mechanisms specific to the new host. For instance, HIV-1 acquired the Vpu gene during its evolution from older SIV ancestors to counteract human tetherin, whereas older SIV-1 ancestors had different counteracting mechanisms specific for simian tetherins [52]. Another example is the species-specific function of the HIV-1 accessory protein Vif, which counteracts APOBEC3G in humans and chimpanzees but not APOBEC3G in monkeys (African green monkeys and rhesus macaques). This human/chimpanzee-specific Vif function is thought to be one reason why HIV-1 can replicate only in humans and chimpanzees, but not in other simians [49]. A similar adaptation may have occurred during PTLV-1 evolution, wherein HTLV-1 acquired (and kept) some features that facilitated better adaptation to infect humans and possibly other apes. We hypothesize that the acquisition of accessory proteins was one such adaptation. In this study, we found different replication patterns between STLV-1 Mfu (which lacks all three accessory proteins) and HTLV-1a (which has them all) in Jurkat T cells and THP-1 myeloid cells, suggesting that the acquisition of these proteins conferred better fitness in the new human host. The assumption that the acquisition of accessory proteins confers a significant selective advantage is supported by the previous observation of spontaneous mutation reversion in HTLV-1 p30 and HBZ knockout mutants injected into rhesus macaques [31]. This spontaneous reversion demonstrates HTLV-1’s ability to rapidly evolve into more competent genomic variants.

Other studies have shown that the accessory proteins are dispensable for the development of HAM/TSP or ATL [25,37,38]. We argue that these proteins are probably not essential for HTLV-1 pathogenesis; however, their emergence made the virus more “adaptable” or “fit” for human-to-human transmission. This may explain why the HTLV-1a subtype is far more prevalent in humans than other subtypes that lack these proteins. In fact, the concept that a virus’s adaptability to humans plays a pivotal role in determining global dominance over others is well established. For instance, during HIV-1 evolution, three groups (M, N, and O) emerged from three independent cross-species transmission events, yet only group M evolved a Vpu protein that was fully adapted to the human host. This observation is thought to explain why group M HIV-1 is responsible for the global HIV/AIDS pandemic [53,54].

Our investigation into HBZ/SBZ functionality across different PTLV-1 subtypes revealed several critical insights. Notably, we found a divergence in HBZ/SBZ sequence length, particularly the acquisition of a shorter HBZ/SBZ sequence by the cosmopolitan and African subtypes in contrast to the longer HBZ/SBZ sequence in HTLV-1c and Asian STLV-1. Here we show that HBZ protein from cosmopolitan HTLV-1a and African STLV-1 subtypes (STLV-1 Ptr, STLV-1 Csa, and STLV-1 Pan) activate the TGF-β/Smad signaling pathway robustly compared to its counterparts from HTLV-1c and Asian STLV-1. sHBZ protein can activate the TGF-β/Smad signaling pathway through its activation domain (AD), while the bZIP domain exhibits a suppressive effect [55]. Specifically, deletion of residues 20–38, which includes the LXXLL-like motif (LXXLL1), abolished TGF-β activation, and mutation of this motif completely eliminated this activity. Our sequence alignment of HBZ/SBZ proteins reveals that a conserved glutamic acid at position 22 in cosmopolitan HTLV-1 is replaced by aspartic acid in African and Asian PTLV-1 (E22D). However, this substitution appears to have no functional impact, as both cosmopolitan HTLV-1 and African PTLV-1 activate the TGF-β/Smad pathway at a comparable level. At position 37, Asian PTLV-1 has a unique glutamine (Q37ins) that is absent in cosmopolitan and African PTLV-1. Notably, the key residues in the LXXLL1 motif are highly conserved across PTLV-1 subtypes, with the exception of a highly conserved glutamic acid at positions 31–33 in cosmopolitan and African PTLV-1 which is replaced by aspartic acid in many Asian PTLV-1 (E31-33D). These differences might contribute to the observed variation in the ability to activate the TGF-β/Smad signaling pathway.

Previously we reported that in HTLV-1a, HBZ protein does not inhibit the deaminase activity of human APOBEC3G, whereas both HBZ and Japanese macaque SBZ inhibit the enzymatic activity of APOBEC3G from Japanese macaques [45]. However, HBZ utilizes human APOBEC3G to synergistically activate the TGF-β/Smad pathway and promote the proliferation of ATL cells, indicating that the combination of HBZ with human APOBEC3G is implicated in the pathogenesis of HTLV-1. Similarly, we found that SBZ from STLV-1 strains found in baboon and to a lesser extent in chimpanzee can synergize with APOBEC3G from these species to activate the TGF-β/Smad signaling pathway. These results suggest that the greater TGF-β activation by HTLV-1a and African STLV-1 subtypes might explain their known pathogenicity in humans, gorillas [2], African green monkeys [4,5], and baboons [3]. Therefore, the evolution of HTLV-1a in the human host may have selected for HBZ mutants with a strengthened ability to manipulate the TGF-β/Smad signaling pathway to promote clonal expansion. However, the inability of the HTLV-1a subtype to inhibit the deaminase activity of APOBEC3G [45] – and likely the African PTLV-1 subtypes – suggests either that they have not yet evolved a mechanism to counteract this host restriction factor or that their interactions with it represent a trade-off, prioritizing the proliferation of the infected cells via the activation of the TGF-β/Smad pathway over the complete evasion of APOBEC3G-mediated editing. However, these findings are based on human cell lines and should be interpreted with caution. While HepG2 cells may not fully reflect T-cell biology, the primary target of PTLV-1 infection, they provide a well-established model for studying TGF-β signaling due to their high transfection efficiency and well-characterized responses. Nevertheless, these observations are highly relevant to understanding the potential pathogenesis of these strains in humans and their respective host species. Whether the observed patterns extend to SBZ and APOBEC3G in their natural host species remains unclear. Future studies using non-human primate models will be essential to determine the role of SBZ and APOBEC3G in modulating TGF-β signaling across species, providing deeper insights into PTLV-1 persistence and pathogenesis.

In summary, this study characterizes the evolution of the accessory and regulatory proteins in PTLV-1 subtypes. Our findings suggest that acquiring the accessory proteins during PTLV-1 evolution in great apes, including humans, potentially aid the virus in adapting to these hosts following interspecies transmission. In addition, HBZ/SBZ proteins show varying capacities to activate the TGF-β/Smad signaling pathway, and this difference may be related to the virulence of PTLV-1 in each host. Understanding these evolutionary differences can help in understanding the mechanisms underlying PTLV-1 pathogenicity and host specificity, and how certain viral strains maintain higher pathogenic potential.

## Materials and methods

### Ethics statement

All human blood samples were collected with written informed consent in accordance with the principles in the Declaration of Helsinki after approval by The Institutional Ethics Committee of Kumamoto University (approval number Genome 297). Japanese macaque (*Macaca fuscata*) peripheral blood mononuclear cells (PBMCs) were collected in accordance with the guidelines and regulations approved by The Animal Welfare and Animal Care Committees of Kyoto University (approval numbers R12-01, R13-01, R14-01, and 2020–101). Frozen PBMCs from Rhesus macaque (*Macaca mulatta*), cynomolgus monkeys (*Macaca fascicularis*), and African green monkeys (*Chlorocebus sabaeus*) were kindly provided by the Center For the Evolutionary Origins Of Human Behavior, Kyoto University, Japan.

### Cells

*Macaca nemestrina* peripheral blood lymphocyte cell line (HSMn-3942) was provided by Dr. Hirofumi Akari, Kyoto University, Japan). The PTLV-1-infected cell lines used in this study included ChM1141 (*Pan troglodytes*) and GM0650 (*Chlorocebus sabaeus*) (both provided by Dr. Tomoyuki Miura, Kyoto University, Japan) and Si-2 (*Macaca fuscata*) from JCRB cell bank (JCRB1321). The PTLV-1 negative cell lines used in this study included Jurkat (provided by Dr. Shimon Sakaguchi, Kyoto University, Japan), THP-1 (provided by Dr. Yoshio Koyanagi, Kyoto University, Japan), HEK293T (ATCC), and HepG2 (provided by Dr. Kunitada Shimotohno, Kyoto University, Japan).

All cells were maintained in RPMI or DMEM supplemented with 10–20% (vol/vol) fetal bovine serum (FBS) and 1% (vol/vol) penicillin/streptomycin solution. GM0650, Si-2, and HSMn-3942 cells were supplemented with recombinant human interleukin 2 (rhIL-2) at 100 U/ml (PeproTech).

### Plasmids

The expression vectors for p12 from HTLV-1a and STLV-1 Ptr (pGFP-2A-h.p12 and pGFP-2A-c.p12, respectively) were constructed by cloning the 2A peptide sequence derived from porcine teschovirus-1 (GGA AGC GGA GCT ACT AAC TTC AGC CTG CTG AAG CAG GCT GGA GAC GTG GAG GAG AAC CCT GGA CCT), the HA peptide and the coding sequence of p12 (human or chimpanzee) in-frame downstream of GFP in a pEGFP-C1 backbone vector. The expression of cleaved HA-p12 was confirmed by immunoblotting using an anti-HA antibody (Sigma). The pX1MT-M infectious molecular clone was kindly provided by David Derse [42].

The expression plasmids pcDNA3.1-FLAG-HsaA3G (*Homo sapiens*; 373aa, or 384aa), -PtrA3G (*Pan troglodytes*; 384aa), -PanA3G (*Papio anubis*; 376aa), -CsaA3G (*Chlorocebus sabaeus*; 376aa, 383aa, or 394aa), -MmuA3G (*Macaca mulatta*; 395aa), -MfaA3G (*Macaca fascicularis*, 394), -MfuA3G (*Macaca fuscata*; 395aa), and -MneA3G (*Macaca nemestrina,* 383aa or 394aa) were constructed by cloning the respective target sequences from PBMCs or cell lines. Briefly, total RNA was extracted using the ReliaPrep RNA miniprep system (Promega) and reverse-transcribed to cDNA with the SuperScript IV reverse transcriptase kit (ThermoFisher Scientific) following the manufacturer’s instructions. The target genes were then amplified using nested PCR with PrimeSTAR HS DNA Polymerase (Takara). The amplified products were cloned into the pcDNA3.1-FLAG plasmid using EcoRI and XbaI restriction enzymes (New England Biolabs), and the final construct was confirmed using Sanger sequencing.

Plasmids pcDNA3.1/myc-His-HTLV-1a HBZ, -STLV-1Ptr SBZ, and -STLV-1Mfu SBZ were generated by cloning HBZ/SBZ sequences from human, chimpanzee, and Japanese macaque samples, respectively. In contrast, plasmids pcDNA3.1/myc-His-(HTLV-1c HBZ), - STLV-1Pan SBZ, - STLV-1Csa SBZ, and – STLV-1Mne SBZ were generated by cloning the full-length synthesized HBZ/SBZ sequences into the pcDNA3.1/myc-His plasmid vector using EcoRI and BamHI restriction enzymes (New England Biolabs). The 3TP-Lux construct and the phRL-TK plasmid were obtained from Promega.

### Provirus sequencing

To determine the full sequence of the STLV-1 provirus, multiple PCR fragments were amplified from genomic DNA to cover the full genome using high-fidelity PrimeSTAR HS DNA Polymerase (Takara). Direct sequencing of these fragments was done by the Sanger method.

### Construction of a full-length STLV-1 molecular clone

Genomic DNA was extracted from PBMCs of an STLV-1-infected Japanese macaque. Multiple overlapping fragments from the STLV-1 provirus were amplified by PCR. These fragments were serially cloned one-by-one into the backbone vector pCR-Blunt II-TOPO (Invitrogen) using unique restriction sites in the provirus sequence. The resulting plasmid was named PCR-Blunt-STLV-1, and its full-length sequence was confirmed by Sanger sequencing.

### Measurement of MHC-I expression

2x10^5^ Jurkat cells were transfected with 2 µg of p12-expressing plasmid by electroporation using Neon Transfection System (Life Technologies) according to the manufacturer’s protocol. At day 5 post-transfection, cells were collected and stained with Pacific Blue labeled anti-human HLA-A, B, and C antibodies (BioLegend) and subjected to FACS analysis using a FACSVerse.

### ELISA for HTLV-1/STLV-1 p19

For titration of virus production, HEK293T cells were seeded in a 12-well plate at a density of 2x10^5^ cells/well. On the next day, the HTLV-1 (pX1MT-M) or STLV-1 (PCR-Blunt-STLV-1) molecular clone was transfected into HEK293T cells using TransIT-LT (Mirus). Seventy-two hours later, the supernatant was collected, and p19 was measured by HTLV p19 Antigen ELISA (ZeptoMetrix).

### Co-culture infectivity

Initially (Day 0), HEK293T cells were seeded in a 24-well plate at a density of 2x10^5^ cells/well in 0.5 ml of DMEM supplemented with 10% FBS. 24 hours later (Day 1), these donor cells were transfected with 0.5 µg of the HTLV-1 or STLV-1 full-length molecular clone using PEI. At Day 3, 2x10^5^ Jurkat or THP-1 recipient cells were transfected with 500 ng of the Firefly HTLV-1 reporter plasmid WT-Luc [56] and 20 ng of the Renilla control plasmid phRL-TK (Promega) using Lipofectamine LTX (Promega). On Day 4, the recipient Jurkat or THP-1 cells were co-cultured with the infectious clone transfected HEK293T cells for another 48 hours, after which the cells were harvested. A luciferase assay was performed using the Dual-Luciferase Reporter Assay System (Promega) according to the manufacturer’s instructions.

To evaluate viral production in Jurkat and THP-1 cells, HEK293T cells were first transfected with either the HTLV-1 or STLV-1 full-length molecular clone. After 48 hours, CFSE-labeled Jurkat or THP-1 recipient cells were co-cultured with the transfected HEK293T cells for an additional 48 hours. The labeled cells were then sorted using Sony SH800S Cell Sorter, transferred to a new plate, and supplemented with fresh complete culture medium. After one week, the culture supernatant was collected and analyzed for viral titers using HTLV p19 Antigen ELISA (ZeptoMetrix).

### Luciferase assay

The 3TP-Lux luciferase assay was performed according to [45]. Briefly, HepG2 cells (1 × 10^5^ per well) were seeded into a 24-well plate. After 24 hours, the cells were transfected with the 3TP-Lux and phRL-TK plasmids, along with the specified plasmid (e.g., pME18S-HBZ/SBZ or pcDNA3.1-FLAG-HsaA3G). The total plasmid DNA levels were kept consistent by using an empty vector. Twenty-four hours post-transfection, the cells were treated with 10ng/mL recombinant human TGF-β1 (Wako). After an additional 24 hours, luciferase activity was measured using the Dual-Luciferase Assay System (Promega) according to the manufacturer’s instructions. All experiments were conducted in triplicate.

### Immunoblotting

For Tax expression, HEK293T cells were transfected with either HTLV-1 (pX1MT-M) or STLV-1 (PCR-Blunt-STLV-1) molecular clones using the cationic polymer polyethylenimine (PEI). Seventy-two hours later, cells were harvested and lysed. For the detection of endogenous STLV-1 Tax expression, Si-2 cell lysate was used. The lysates were subjected to immunoblotting using an anti-Tax antibody (MI-73) [57].

For sHBZ/SBZ and APOBEC3G, cells were transfected as described above. The lysate were subjected to immunoblotting using mouse anti-tubulin antibody, goat anti-mouse IgG HRP-linked antibody, anti-Flag IgG HRP-linked antibody, rabbit anti-His antibody, and goat anti-rabbit IgG HRP-linked antibody. The immunoblot data were acquired using the ChemiDoc imaging system (Bio-Rad).

### Phylogenetic analysis of the PTLV-1

PTLV-1 along with two HTLV-2 (outgroup) *Env* sequences (522 bp) (summarized in S1 Table) were aligned using the L-INS-I program in MAFFT version 7.309 [58]. Then maximum likelihood phylogenetic trees were constructed using MEGA11: Molecular Evolutionary Genetics Analysis version 11 software [59,60], with Kimura 2-parameter model [61] and gamma-distributed rates among sites which were selected using the best-fit substitution model in the software. Initial tree(s) for the heuristic search were obtained automatically by applying Neighbor-Join and BioNJ algorithms to a matrix of pairwise distances estimated using the Maximum Composite Likelihood (MCL) approach and then selecting the topology with superior log likelihood value. A discrete Gamma distribution was used to model evolutionary rate differences among sites (5 categories (+G, parameter = 0.2890)).

To examine the possibility of recombination events in the PTLV-1 genomes, we first aligned PTLV-1 genomes (summarized in S4 Table) using the L-INS-i program in MAFFT suites. Based on the alignment, RDP version 3.44 [62] was applied to detect recombination.

### Phylogenetic analysis of APOBEC3G

APOBEC3G sequences from various primate species (summarized in S5 Table) were retrieved from public databases along with *Mus musculus* APOBEC3 protein (outgroup). Additionally, APOBEC3G sequences from the Japanese macaque (Mfu_1188 bp/395aa), which we amplified and sequenced in-house, were included. Sequence alignments were performed using MAFFT online service version 7 [58] with the L-INS-i method. Maximum likelihood phylogenetic trees were constructed with MEGA11 software [59,60], employing the Kimura 2-parameter nucleotide substitution model [61] and gamma-distributed rates among sites, as determined by the best-fit substitution model in the software. The tree with the highest log likelihood (-4636.76) is shown. Initial tree(s) for the heuristic search were obtained automatically by applying Neighbor-Join and BioNJ algorithms to a matrix of pairwise distances estimated using the MCL approach, and then selecting the topology with superior log likelihood value. A discrete Gamma distribution was used to model evolutionary rate differences among sites (5 categories (+G, parameter = 1.5680)). The tree is drawn to scale, with branch lengths measured in the number of substitutions per site [59,60].

### Protein alignment

Coding sequences for accessory and HBZ/SBZ proteins were aligned using MUSCLE [63], and figures were generated using Jalview version 2.11.3.3 [64].

### Prediction of coding potential for pX region of PTLV-1 strains

To predict the coding potential of different PTLV-1 strains, pX region sequences were first aligned using MUSCLE and visualized using the CLC Main Workbench. HTLV-1 subtype A (AF033817) was used as a reference for prediction. For each regulatory/accessory protein, the segment of spliced mRNA that aligned to the coding region of the reference sequence was extracted, and the encoded protein’s sequence was predicted. Coding sequences with premature stop codons (compared with the reference) were considered truncated mutants. Although alternative initiation codons and splicing donor/acceptor sites are possible, we included only the alignments between the reference initiation and stop codons in this analysis.

## Supporting information

S1 Fig Multiple sequence alignment of p12 protein. Translated nucleotide sequences for the p12 reading frame from various PTLV-1 subtypes were aligned using MUSCLE default settings. Jalview software was used for figure production. A dash (-) represents a gap in the sequence alignment. An asterisk (*) in the sequence alignment indicates a stop codon, while an asterisk in the box for conservation represents a fully conserved residue. Numbers under the boxes for conservation represent the conservation score for each column in the alignment (higher numbers indicate higher conservation). The colors are built-in color schemes based on amino acid symbols.
(TIF)

S2 Fig Multiple sequence alignment of p30 protein. Translated nucleotide sequences for the p30 reading frame from various PTLV-1 subtypes were aligned using MUSCLE default settings. Jalview software was used for figure production. A dash (-) represents a gap in the sequence alignment. An asterisk (*) in the sequence alignment indicates a stop codon, while an asterisk in the box for conservation represents a fully conserved residue. Numbers under the boxes for conservation represent the conservation score for each column in the alignment (higher numbers indicate higher conservation). The colors are built-in color schemes based on amino acid symbols.
(TIF)

S3 Fig Multiple sequence alignment of p13 protein. Translated nucleotide sequences for the p13 reading frame from various PTLV-1 subtypes were aligned using the MUSCLE default settings. Jalview software was used for figure production. A Dash (-) represents a gap in the sequence alignment. An asterisk (*) in the sequence alignment indicates a stop codon, while an asterisk in the box for conservation represents a fully conserved residue. Numbers under the boxes for conservation represent the conservation score for each column in the alignment (higher numbers indicate higher conservation). The colors are built-in color schemes based on amino acid symbols.
(TIF)

S4 Fig Percentage of identity and sequence length of PTLV-1 accessory and regulatory proteins. Heatmap showing the percentage of identity and the coding sequence length until a stop codon of the PTLV-1 accessory (p12, p30, p13, and HBZ/SBZ) and regulatory (Tax and Rex) proteins in various PTLV-1 subtypes. HTLV-1a AF033817 was used as a reference sequence.
(TIF)

S5 Fig Multiple sequence alignment of APOBEC3G protein. APOBEC3G protein coding sequences from various primate species were aligned using the MUSCLE default settings. Jalview software was used for figure production.
(TIF)

S1 Table Information for the proviral sequences used for phylogenetic analysis in Fig 1. A partial sequence of the *Env* coding region (522 bp) was used in this analysis. (XLSX)

S2 Table Information for proviral sequences with full-length pX region related to Fig 2. (XLSX)

S3 Table Results for the analysis of the coding potential of pX region ORFs in various strains of HTLV-1/STLV-1. All predictions are based on sites aligning to the annotated sites of interest in the reference sequence (AF033817); alternative sites for splicing or translation initiation were not considered in this analysis. The three dashes represent missing information for ATG initiation sites. The two dashes represent missing information for the splicing donor/acceptor sites. The consensus splice site used is (GT==AG), where “GT” refers to the splice donor site and “AG” refers to the splice acceptor site for each intron in the gene of interest. For example, the consensus splice sites for the Tax gene (which has three exons and two introns) in the AF033817 reference genome are GT==AG (Tax Intron 1) and GT==AG (Tax Intron 2). These results are summarized in Fig 2 and S4 Fig.
(XLSX)

S4 Table Information for proviral sequences used in recombination analysis. (XLSX)

S5 Table APOBEC3(G) sequences used for multiple sequence alignment and phylogenetic analysis. (XLSX)
