# Correction: Evolution of primate T-cell leukemia virus type 1 accessory genes and functional divergence of its antisense proteins

**DOI:** 10.1371/journal.ppat.1013668

**Published:** 2025-11-07

**Authors:** Osama Hussein, Mohamed Mahgoub, Takafumi Shichijo, So Nakagawa, Junko Tanabe, Hirofumi Akari, Tomoyuki Miura, Masao Matsuoka, Jun-ichirou Yasunaga

The Data Availability statement for this article is incorrect. The full-length proviral sequence of STLV-1 isolated from Pan troglodytes, Chlorocebus sabaeus, and Macaca fuscata have been deposited in the NCBI public database (GenBank accession numbers MH542224, MH542225, and MH542226, respectively). Other relevant data underlying the results presented in this study are available from Figshare (DOI: 10.6084/m9.figshare.30094336).
