# Supplementary figures and images for: Evolution of primate T-cell leukemia virus type 1 accessory genes and functional divergence of its antisense proteins
Source: PLoS Pathog. 2025 May 9;21(5):e1013158. doi: 10.1371/journal.ppat.1013158 (PMC12088518; doi:10.1371/journal.ppat.1013158)

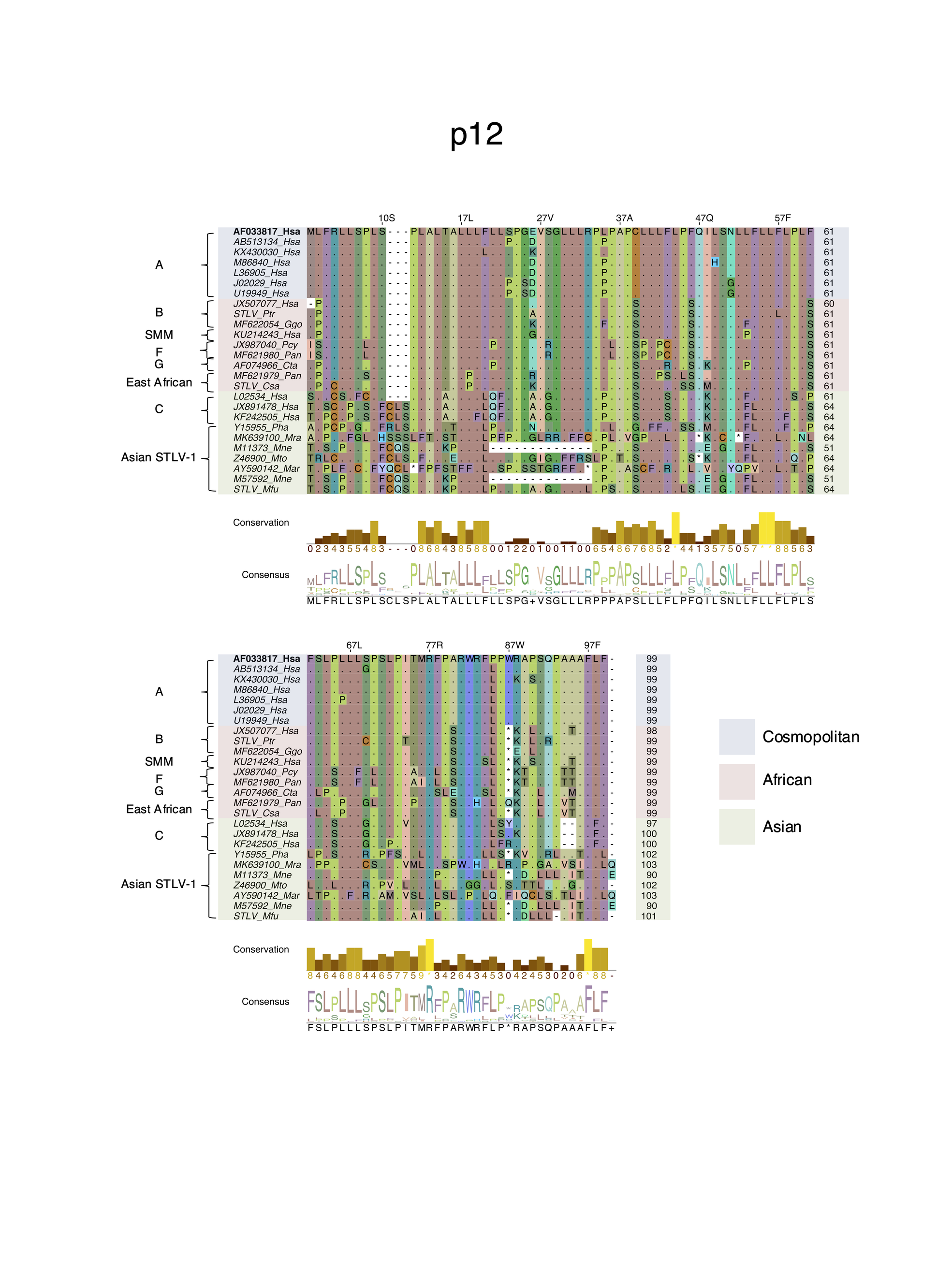

Supplement: S1 Fig — Translated nucleotide sequences for the p12 reading frame from various PTLV-1 subtypes were aligned using MUSCLE default settings. Jalview software was used for figure production. A dash (-) represents a gap in the sequence alignment. An asterisk (*) in the sequence alignment indicates a stop codon, while an asterisk in the box for conservation represents a fully conserved residue. Numbers under the boxes for conservation represent the conservation score for each column in the alignment (higher numbers indicate higher conservation). The colors are built-in color schemes based on amino acid symbols. (TIF) [file ppat.1013158.s001.tif]

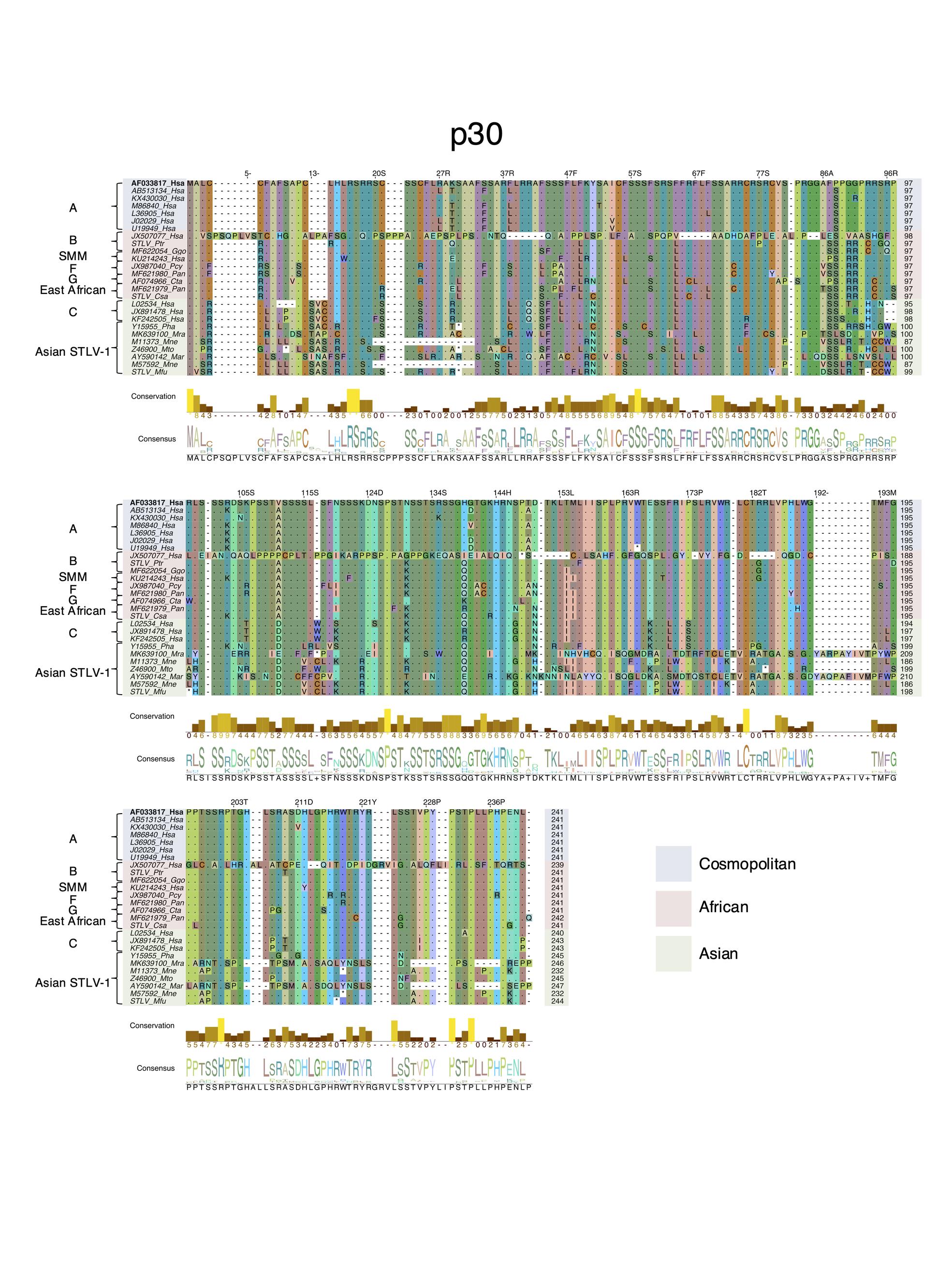

Supplement: S2 Fig — Translated nucleotide sequences for the p30 reading frame from various PTLV-1 subtypes were aligned using MUSCLE default settings. Jalview software was used for figure production. A dash (-) represents a gap in the sequence alignment. An asterisk (*) in the sequence alignment indicates a stop codon, while an asterisk in the box for conservation represents a fully conserved residue. Numbers under the boxes for conservation represent the conservation score for each column in the alignment (higher numbers indicate higher conservation). The colors are built-in color schemes based on amino acid symbols. (TIF) [file ppat.1013158.s002.tif]

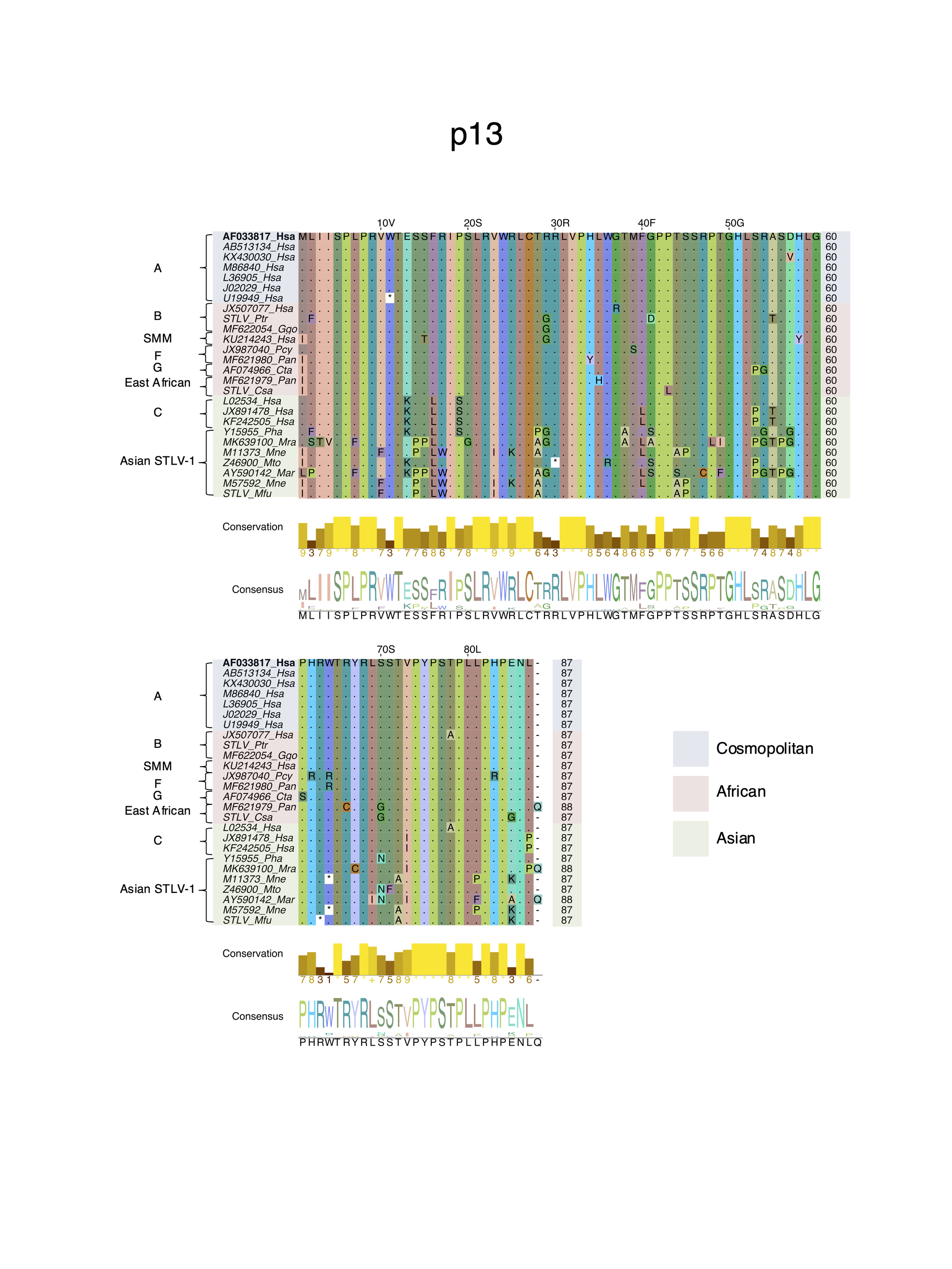

Supplement: S3 Fig — Translated nucleotide sequences for the p13 reading frame from various PTLV-1 subtypes were aligned using the MUSCLE default settings. Jalview software was used for figure production. A Dash (-) represents a gap in the sequence alignment. An asterisk (*) in the sequence alignment indicates a stop codon, while an asterisk in the box for conservation represents a fully conserved residue. Numbers under the boxes for conservation represent the conservation score for each column in the alignment (higher numbers indicate higher conservation). The colors are built-in color schemes based on amino acid symbols. (TIF) [file ppat.1013158.s003.tif]

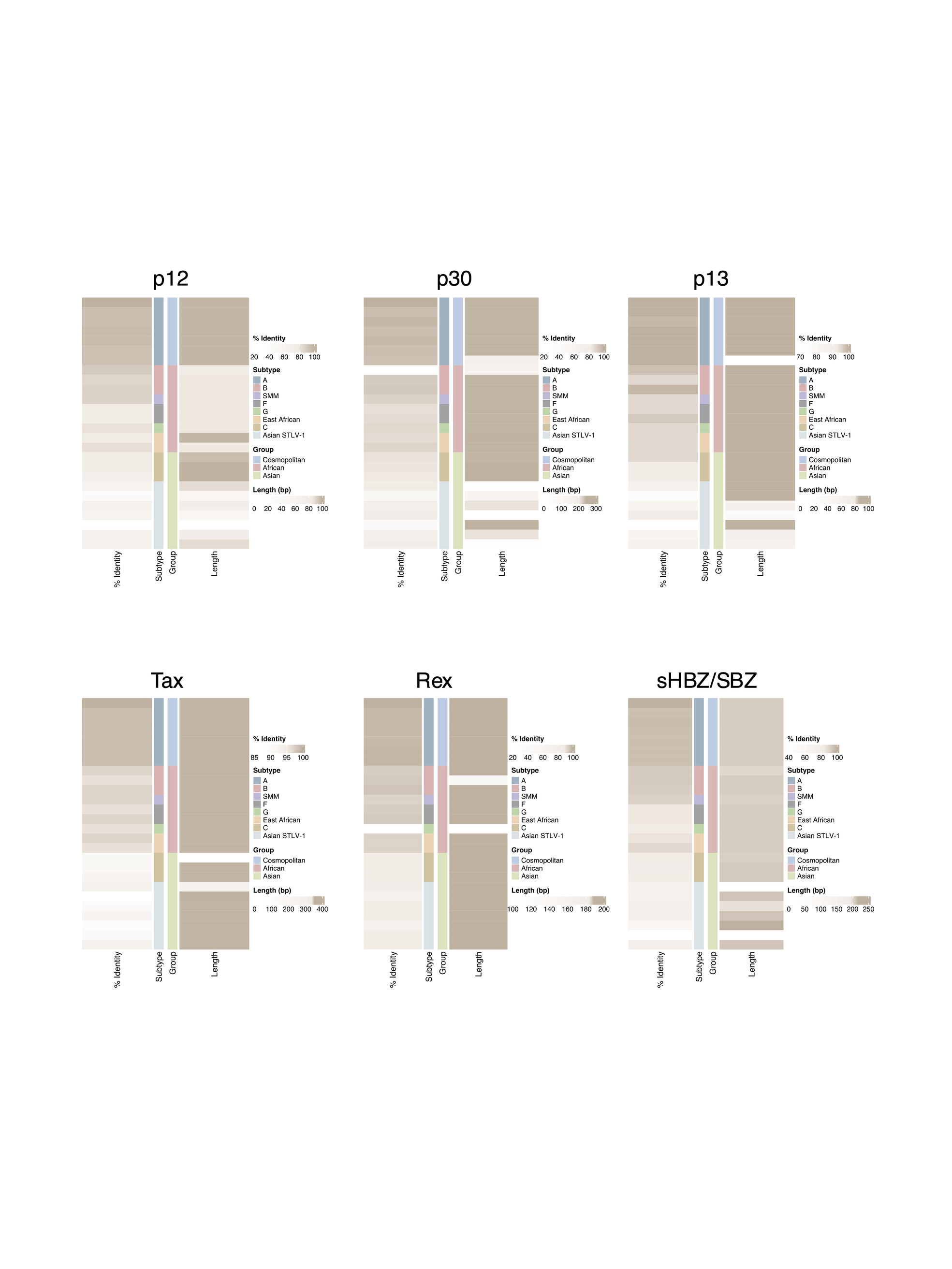

Supplement: S4 Fig — Heatmap showing the percentage of identity and the coding sequence length until a stop codon of the PTLV-1 accessory (p12, p30, p13, and HBZ/SBZ) and regulatory (Tax and Rex) proteins in various PTLV-1 subtypes. HTLV-1a AF033817 was used as a reference sequence. (TIF) [file ppat.1013158.s004.tif]

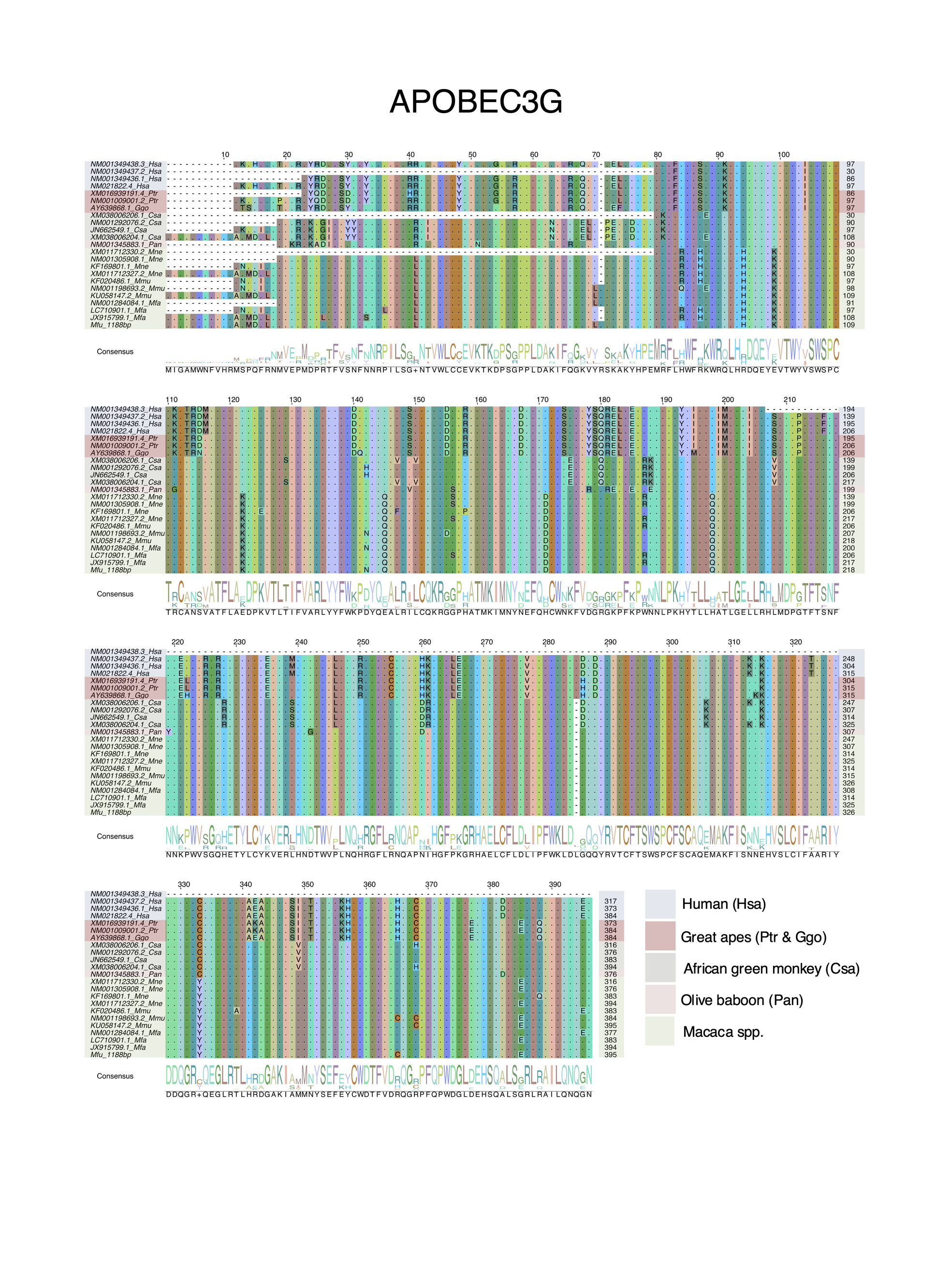

Supplement: S5 Fig — APOBEC3G protein coding sequences from various primate species were aligned using the MUSCLE default settings. Jalview software was used for figure production. (TIF) [file ppat.1013158.s005.tif]
